# Supplementary material for: The Role of Artificial Intelligence in Predicting Outcomes by Cardiovascular Magnetic Resonance: A Comprehensive Systematic Review
Source: Medicina (Kaunas). 2022 Aug 12;58(8):1087. doi: 10.3390/medicina58081087 (PMC9412853; doi:10.3390/medicina58081087)
Supplement: Supplementary file 1 [file medicina-58-01087-s001.zip › medicina-1783189-supplementary.pdf]

Supplementary Table S1. Summary of all CMR parameters and their significance for studies included in this systematic review.

First Author	Disease	CMR Parameter	HR	95% CI	P value
**Dawes et al. (1)**	PH	RV 3D motion	2.75	1.73-4.30	0.001
		RV ESVI	2.09	0.77-5.71	0.149
		SV/ESV	2.08	0.76-5.68	0.152
		RV EDVI	1.87	0.68-5.09	0.221
		RV EF	1.79	0.82-3.87	0.140
		RV EDP	1.71	0.62-4.68	0.292
**Schuster et al. (2)**	MI	LV EF	0.94	0.92-0.96	0.001
		IS	1.05	1.02-1.07	0.001
		MVO	1.07	1.01-1.10	0.016
**Diller et al. (3)**	ToF	RV LAX strain	0.86	0.77-0.96	0.009
		LV LAX max.area	1.09	1.03-1.14	0.002
		RV LAX max.area	1.05	1.00-1.09	0.04
		LV SAX min.area	1.15	1.04-1.27	0.005
		LV SAX strain	0.89	0.80-0.99	0.046
		LV SAX med.area	1.09	1.00-1.19	0.043
		RA min.area	1.10	1.02-1.18	0.01
		RA med. area	1.10	1.03-1.18	0.003
		RA max.area	1.09	1.03-1.16	0.003
		LV SAX max.area	1.09	1.01-1.18	0.02
**Knott et al. (4)**	CAD	Stress MBF	3.02	2.34-3.89	0.001
		MPR	2.40	1.91-3.01	0.001

**Seraphim et al. (5)**	CAD	Rest PTT	1.59	1.31-1.92	0.001
		PBVi	1.46	1.19-1.80	0.001
		Stress PTT	1.17	1.10-1.25	0.001
		LV EF	0.96	0.95-0.98	0.001

Abbreviations: 3D, three-dimensional; CAD, coronary artery disease; CI, confidence interval; EF, ejection fraction; EDP, end-diastolic pressure; EDVI, end-diastolic volume index; ESV, end-systolic volume; ESVI, end-systolic volume index; HR, hazard ratio; IS, infarction size; LAX, long-axis; LV, left ventricle; MBF, myocardial blood flow; MI, myocardial infarction; MPR, myocardial perfusion reserve; MVO, microvascular obstruction; PH, pulmonary hypertension; PTT, pulmonary transit time; PBVi, pulmonary blood volume index; RA, right atrium; RV, right ventricle; SAX, short-axis; SV, stroke volume; ToF, tetralogy of fallot.

Supplementary Table S2. Summary of the CLAIM outcomes for all five assessed studies (6).

**CLAIM Item**	**Dawes et al.** (1)	**Schuster, et al.** (2)	**Diller et al.** (3)	**Knott et al.** (4)	**Seraphim et al.** (5)
1 Identification as a study of AI methodology, specifying the category of technology used (e.g. deep learning)	✓	✓	✓	✓	✓
2 Structured summary of study design, methods, results, and conclusions	✓	✓	✓	✓	✓
3 Scientific and clinical background, including the intended use and clinical role of the AI approach	✓	✓	✓	✓	✓

4 Study objectives and hypotheses	✓	✓	✓	✓	✓
5 Prospective or retrospective study	✓	✓	✓	✓	✓
6 Study goal, such as model creation, exploratory study, feasibility study, noninferiority trial	✓	✓	✓	✓	✓
7 Data sources	✓	✓	✓	✓	✓
8 Eligibility criteria: how, where, and when potentially eligible participants or studies were identified (e.g. symptoms, results from previous tests, inclusion in registry, patient- care setting, location, dates)	✓	✓	✓	✓	✓
9 Data preprocessing steps	✓	✓	✓	✓	✓
10 Selection of data subsets, if applicable	✓	✓	✓	✓	✓
11 Definitions of data elements, with references to common data elements	✓	✓	✓	✓	✓
12 De- identification methods	✗	✗	✗	✗	✗
13 How missing data were handled	✗	✓	✓	✓	✗
14 Definition of ground truth reference standard, in sufficient detail to allow replication	✓	✓	✓	✓	✓
15 Rationale for choosing the reference standard (if alternatives exist)	✓	✓	✓	✓	✓
16 Source of ground truth annotations; qualifications and preparation of annotators	✓	✓	✓	✓	✗
17 Annotation tools	✓	✓	✓	✓	✓

18 Measurement of inter- and intrarater variability; methods to mitigate variability and/or resolve discrepancies	✓	✓	✓	✓	✓
19 Intended sample size and how it was determined	✓	✓	✓	✓	✓
20 How data were assigned to partitions; specify proportions	✓	✓	✓	✓	✓
21 Level at which partitions are disjoint (e.g. image, study, patient, institution)	✓	✓	✓	✓	✓
22 Detailed description of model, including inputs, outputs, all intermediate layers and connections	✓	✓	✓	✓	✓
23 Software libraries, frameworks, and packages	✓	✓	✓	✓	✓
24 Initialization of model parameters (e.g. randomization, transfer learning)	✓	✓	✓	✓	✓
25 Details of training approach, including data augmentation, hyperparameters, number of models trained	✓	✓	✓	✓	✓
26 Method of selecting the final model	✓	✓	✓	✓	✓
27 Ensembling techniques, if applicable	✓	✓	✓	✓	✓
28 Metrics of model performance	✓	✓	✓	✓	✓
29 Statistical measures of significance and uncertainty (e.g. confidence intervals)	✓	✓	✓	✓	✓
30 Robustness or sensitivity analysis	✓	✗	✗	✓	✓

31 Methods for explainability or interpretability (e.g. saliency maps) and how they were validated	✓	✓	✓	✓	✓
32 Validation or testing on external data	✓	✓	✓	✓	✓
33 Flow of participants or cases, using a diagram to indicate inclusion and exclusion	✓	✓	✗	✓	✓
34 Demographic and clinical characteristics of cases in each partition	✓	✓	✓	✓	✓
35 Performance metrics for optimal model(s) on all data partitions	✓	✓	✓	✓	✓
36 Estimates of diagnostic accuracy and their precision (such as 95% confidence intervals)	✓	✓	✓	✓	✓
37 Failure analysis of incorrectly classified cases	✓	✓	✓	✓	✓
38 Study limitations, including potential bias, statistical uncertainty, and generalizability	✓	✓	✓	✓	✓
39 Implications for practice, including the intended use and/or clinical role	✓	✓	✓	✓	✓
40 Registration number and name of registry	✗	✓	✓	✓	✓
41 Where the full study protocol can be accessed	✓	✓	✓	✓	✓
42 Sources of funding and other support; role of funders	✓	✓	✓	✓	✓
Total	39	40	39	41	39

Supplemental Figure S1. Forest plot of AI using CMR parameters and their significance of automated volumetric, functional and area assessment in tetralogy of fallot (ToF).

First Author	Disease	CMR parameter	HR	95% CI	P-value
Diller et al.	ToF	RV LAX strain	0.86	0.77-0.96	0.009
Diller et al.	ToF	LV LAX max.area	1.09	1.03-1.14	0.002
Diller et al.	ToF	RV LAX max.area	1.05	1.00-1.09	0.04
Diller et al.	ToF	LV SAX min.area	1.15	1.04-1.27	0.005
Diller et al.	ToF	LV SAX strain	0.89	0.80-0.99	0.046
Diller et al.	ToF	LV SAX med.area	1.09	1.00-1.19	0.043
Diller et al.	ToF	RA min. area	1.10	1.02-1.18	0.01
Diller et al.	ToF	RA med. area	1.10	1.03-1.18	0.003
Diller et al.	ToF	RA max. area	1.09	1.03-1.16	0.003
Diller et al.	ToF	LV SAX max.area	1.09	1.01-1.18	0.02

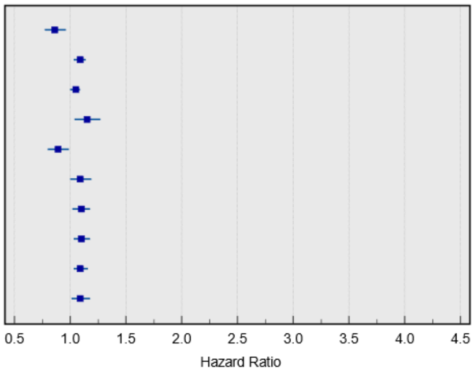

Supplemental Appendix SA. Database search strategy—18/11/2021

Medline and Embase

1 ((deep or supervised or unsupervised or machine) and learning).mp.

[mp=ti, ab, hw, tn, ot, dm, mf, dv, kf, fx, dq, nm, ox, px, rx, ui, sy] (204587)

2 exp "ARTIFICIAL INTELLIGENCE"/ (182929)

3 exp "MACHINE LEARNING"/ (314929)

4 exp "DEEP LEARNING"/ (27686)

5 exp "CONVOLUTION ALGORITHM"/ (12148)

6 exp "DEEP NEURAL NETWORK"/ (15061)

7 (heart or cardi* or myocard* or coronar* or ventric* or LV or RV).mp.

[mp=ti, ab, hw, tn, ot, dm, mf, dv, kf, fx, dq, nm, ox, px, rx, an, ui, sy] (6442532)

8 (Cine or LGE or gadolinium or MRI or MRA or (Magnetic and resonance)).

mp. [mp=ti, ab, hw, tn, ot, dm, mf, dv, kf, fx, dq, nm, ox, px, rx, an, ui, sy] (2426984)

9	exp "CARDIOVASCULAR MAGNETIC RESONANCE"/	(43211)
10	exp "MAGNETIC RESONANCE IMAGING"/	(1564866)
11	8 or 9 or 10	(2445848)
12	(prognos* or predict* or clinical* or outcome* or associa* or risk* or death or mortal* or surviv* or follow-up or course or progress* or deteriorat* or worse*).mp. [mp=ti, ab, hw, tn, ot, dm, mf, dv, kf, fx, dq, nm, ox, px, rx, an, ui, sy]	(34016253)
13	1 or 2 or 3 or 4 or 5 or 6	(495773)
14	7 and 11 and 12 and 13	(2475)
15	limit 14 to english language	(2459)
16	limit 15 to human	(2158)
17	limit 16 to humans	(2158)
18	limit 17 to yr="2021"	(453)
19	remove duplicates from 18	(389)
20	remove conference abstracts	(251)

References

1. Dawes TJW, de Marvao A, Shi W, Fletcher T, Watson GMJ, Wharton J, et al. Machine Learning of Three-dimensional Right Ventricular Motion Enables Outcome Prediction in Pulmonary Hypertension: A Cardiac MR Imaging Study. Radiology. 2017 May;283(2):381–90.

2. Schuster A, Lange T, Backhaus SJ, Strohmeyer C, Boom PC, Matz J, et al. Fully Automated Cardiac Assessment for Diagnostic and Prognostic Stratification Following Myocardial Infarction. J Am Heart Assoc Cardiovasc Cerebrovasc Dis. 2020 Sep 2;9(18):e016612.

3. Diller GP, Orwat S, Vahle J, Bauer UMM, Urban A, Sarikouch S, et al. Prediction of prognosis in patients with tetralogy of Fallot based on deep learning imaging analysis. Heart. 2020 Jul 1;106(13):1007–14.

4. Knott KD, Seraphim A, Augusto JB, Xue H, Chacko L, Aung N, et al. The Prognostic Significance of Quantitative Myocardial Perfusion. Circulation. 2020 Apr 21;141(16):1282–91.

5. Seraphim A, Knott KD, Menacho K, Augusto JB, Davies R, Pierce I, et al. Prognostic Value of Pulmonary Transit Time and Pulmonary Blood Volume Estimation Using Myocardial Perfusion CMR. Jacc Cardiovasc Imaging. 2021 Nov;14(11):2107–19.

6. Mongan J, Moy L, Kahn CE. Checklist for Artificial Intelligence in Medical Imaging (CLAIM): A Guide for Authors and Reviewers. Radiol Artif Intell. 2020 Mar 1;2(2):e200029.
